# Can ChatGPT Be Considered an Author of a Medical Article?

**DOI:** 10.2188/jea.JE20230030

**Published:** 2023-07-05

**Authors:** Kazuki Ide, Philip Hawke, Takeo Nakayama

**Affiliations:** 1 Division of Scientific Information and Public Policy, Center for Infectious Disease Education and Research (CiDER), Osaka University, Osaka, Japan; 2 Research Center on Ethical, Legal and Social Issues, Osaka University, Osaka, Japan; 3 National Institute of Science and Technology Policy (NISTEP), Tokyo, Japan; 4 School of Pharmaceutical Sciences, University of Shizuoka, Shizuoka, Japan; 5 Department of Health Informatics, Graduate School of Medicine/School of Public Health, Kyoto University, Kyoto, Japan

The technology of generative artificial intelligence (AI) is developing rapidly, and recently there has been great interest in the chatbot ChatGPT, released by OpenAI (San Francisco, CA, USA) in November 2022.^1^ Its high performance is evidenced by the fact that it scored at or near the passing standard on the United States Medical Licensing Exam (USMLE),^2^ and its potential implementation in healthcare is now under discussion in the United States.^3^ It has also been reported to be difficult to distinguish between abstracts generated by ChatGPT and those written by humans, with scientists mistaking 32% of ChatGPT abstracts as being human produced.^4^

These developments raise the issue of whether ChatGPT is capable of true authorship, especially as ChatGPT has already been named as a co-author of at least four scientific papers, including some in the fields of medicine and nursing.^5^ To clarify this issue, we assessed whether ChatGPT actually meets the criteria for authorship of a medical article based on the guidelines of the International Committee of Medical Journal Editors (ICMJE). The ICMJE author criteria are as follows^6^:

1. Substantial contributions to the conception or design of the work; or the acquisition, analysis, or interpretation of data for the work; AND
2. Drafting the work or revising it critically for important intellectual content; AND
3. Final approval of the version to be published; AND
4. Agreement to be accountable for all aspects of the work in ensuring that questions related to the accuracy or integrity of any part of the work are appropriately investigated and resolved.

In order to provide a timely assessment of these criteria in the rapidly changing context of AI development, the criteria were reviewed and assessed by the two specialists involved in the present research project (KI and TN), then further discussed with the other contributor (PH). The results of this analysis are shown in Table 1, outlining the extent to which ChatGPT fulfills the criteria. The table reveals that, depending on the user’s prompt, ChatGPT can fulfill criteria 1 and 2, but also that ChatGPT cannot fulfill criteria 3 and 4. Of course, it is possible that the ICMJE will change its authorship criteria in response to developments in AI. For example, as ChatGPT appears to be helpful for increasing the productivity of authors, the organization may allow the inclusion of ChatGPT as a co-author to allow readers to easily find articles that have used it. However, such changes would still not alter the fact that ChatGPT at present does not appear to be capable of thinking sufficiently independently to fulfill criteria 3 and 4, giving final approval to and being accountable for the work.

**Table 1.** Does ChatGPT meet the authorship criteria of the International Committee of Medical Journal Editors?

Criterion number	Criterion content	Yes	No
1	Substantial contributions to the conception or design of the work; or the acquisition, analysis, or interpretation of data for the work	✓	
2	Drafting the work or revising it critically for important intellectual content	✓	
3	Final approval of the version to be published		✓
4	Agreement to be accountable for all aspects of the work in ensuring that questions related to the accuracy or integrity of any part of the work are appropriately investigated and resolved		✓

Based on these considerations, we conclude that it is inappropriate for ChatGPT to be named as an author, at least in journals that have adopted the ICMJE criteria. Nevertheless, we emphasize that it is essential for the transparency of any study using ChatGPT to clearly mention its use in the study’s acknowledgments. The ICMJE may consider adding such principles to its recommendations in its “Non-Author Contributors” section.^6^ This suggestion is in line with the view of the World Association of Medical Editors that chatbots cannot be authors and that, if they are used, the paper’s authors should be transparent about this usage and take responsibility for the content produced by the chatbots.^7^

In response to our prompt “Can you be a co-author of a medical article?”, ChatGPT itself gave the following answer, which is consistent with our view: “As a language model, I am not able to be a co-author of a medical article because I am not a human and do not have the ability to conduct research or contribute original ideas. However, I can assist with the writing and editing of an article. It is important to note that any information generated by me should be fact-checked and independently verified by a qualified professional before being used in any formal research or publication.”

The Science Family of Journals has already gone much further than this in restricting the use of AI in the articles it will accept for publication. It recently updated its editorial policies as follows^8^^,^^9^:

Artificial intelligence (AI) policy: Text generated from AI, machine learning, or similar algorithmic tools cannot be used in papers published in Science journals, nor can the accompanying figures, images, or graphics be the products of such tools, without explicit permission from the editors. In addition, an AI program cannot be an author of a Science journal paper. A violation of this policy constitutes scientific misconduct.

While we think that such a strict policy will certainly help to maintain authorial transparency, we are also concerned that it may overly strict, prematurely preventing researchers from benefiting from the enhanced productivity that AI promises.

It is important for humanity to consider from an early stage how to adopt AI technologies both practically and ethically in order to creatively advance scientific research, including research in epidemiology. Such discussions would be meaningful for us to become more creative in co-creation with AI.
